# Natural selection governs local, but not global, evolutionary gene coexpression networks in *Caenorhabditis elegans*

**DOI:** 10.1186/1752-0509-2-96

**Published:** 2008-11-13

**Authors:** I King Jordan, Lee S Katz, Dee R Denver, J Todd Streelman

**Affiliations:** 1 School of Biology, Georgia Institute of Technology, Atlanta, GA, USA; 2 Department of Zoology & Center for Genome Research and Biocomputing, Oregon State University, Corvallis, OR, USA; 3 School of Biology & Institute of Bioengineering and Bioscience, Georgia Institute of Technology, Atlanta, GA, USA

## Abstract

**Background:**

Large-scale evaluation of gene expression variation among *Caenorhabditis elegans *lines that have diverged from a common ancestor allows for the analysis of a novel class of biological networks – evolutionary gene coexpression networks. Comparative analysis of these evolutionary networks has the potential to uncover the effects of natural selection in shaping coexpression network topologies since *C. elegans *mutation accumulation (MA) lines evolve essentially free from the effects of natural selection, whereas natural isolate (NI) populations are subject to selective constraints.

**Results:**

We compared evolutionary gene coexpression networks for *C. elegans *MA lines versus NI populations to evaluate the role that natural selection plays in shaping the evolution of network topologies. MA and NI evolutionary gene coexpression networks were found to have very similar global topological properties as measured by a number of network topological parameters. Observed MA and NI networks show node degree distributions and average values for node degree, clustering coefficient, path length, eccentricity and betweeness that are statistically indistinguishable from one another yet highly distinct from randomly simulated networks. On the other hand, at the local level the MA and NI coexpression networks are highly divergent; pairs of genes coexpressed in the MA versus NI lines are almost entirely different as are the connectivity and clustering properties of individual genes.

**Conclusion:**

It appears that selective forces shape how local patterns of coexpression change over time but do not control the global topology of *C. elegans *evolutionary gene coexpression networks. These results have implications for the evolutionary significance of global network topologies, which are known to be conserved across disparate complex systems.

## Background

The regulation of genes, resulting in specific patterns and levels of mRNA expression, is thought to be critically important for cellular function, organismal development and evolution. Recent studies have shown that while expression of some genes may change rapidly within and between species [1-3], the topological properties of gene coexpression networks are substantially conserved [4-6]. In other words, the time, place and level at which genes are expressed may be highly dynamic and flexible, but the way that thousands of expression patterns are organized into complex networks seems nevertheless constrained.

The emergent topological similarity among diverse biological networks has suggested to some that 'topology matters' and that natural selection is the evolutionary force governing the pattern [7]. This has galvanized competing perspectives on whether [7] or not [8] the elucidation of the global topological properties of biological networks can yield meaningful insight about local cellular functions and evolution. We conducted an evaluation of the role of natural selection in the evolutionary conservation of gene coexpression networks to address this outstanding issue.

**Figure 1:**
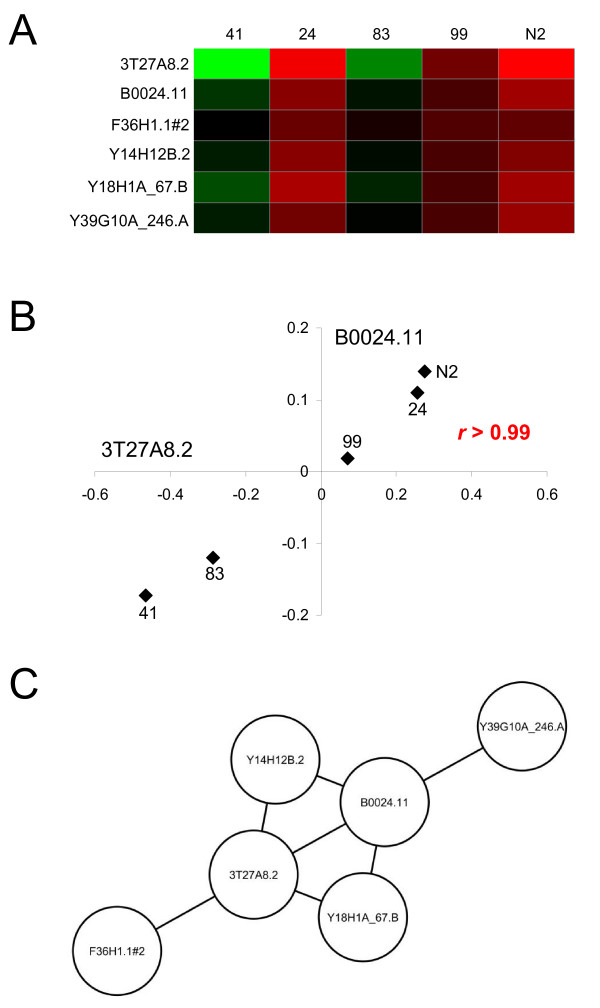
**Evolutionary gene coexpression networks**. (A) Gene expression levels were measured across lines of *C. elegans *that diverged from a common ancestor. Relative expression levels across five MA lines (41, 24, 83, 99, N2) are shown for six genes. (B) Relative levels of gene expression across lines were compared for all pairs of genes using the Pearson correlation coefficient as shown here for genes B0024.11 × 3T27AS8.2. (C) In the evolutionary gene coexpression networks, genes are represented as nodes and gene pairs with *r*-values above the threshold cut-off are linked by edges.

Determination of the effects of natural selection on network topologies was accomplished through the analysis of a novel class of gene coexpression networks that are distinct from the more familiar coexpression networks based on changes in expression over developmental time, tissue or experimental treatment. We refer to these distinct networks as 'evolutionary gene coexpression networks' to reflect the fact that they are based on measures of how gene expression changes over evolutionary time. To build evolutionary gene coexpression networks, variation in levels of gene expression were measured across lines (populations) of *Caenorhabditis elegans *that have diverged from a common ancestor (Figure 1A). Pairs of genes, represented as nodes, that show coordinated changes in expression across lines are linked by edges to form evolutionary gene coexpression networks (Figure 1B &1C). Evolutionary genetic variation in regulation among lines is required to observe correlations between patterns of gene expression, and pairs of genes may be coexpressed across populations because they are regulated by shared factors, cis or trans, that have changed over time. For instance, it was previously noted that *C. elegans *genes differentially expressed across lines are enriched for specific functional categories, chromosomal locations and gene coexpression mounts [9].

Elucidation of the role that natural selection plays in shaping evolutionary gene coexpression network topologies was made possible through the comparison of gene expression patterns among *C. elegans *populations that evolved under a regime of natural selection versus those that evolved in the virtual absence of selection. Previously, *C. elegans *mutation accumulation (MA) lines were bred in order to produce populations that evolve effectively free from selective constraint [10]. Microarray analysis has been used to compare gene expression levels for thousands of *C. elegans *genes among such MA lines with orthologous gene expression for natural isolate (NI) populations [9]. This study clearly demonstrated a role for natural selection in constraining expression divergence, since a much higher fraction of MA than NI genes were found to be differentially expressed across populations. Other studies have demonstrated selective constraint on changes in gene expression between mammals [11-13] and in the fly [14]. We wanted to evaluate how selective constraint on gene expression is manifest in the topologies of evolutionary gene coexpression networks. Given that *C. elegans *MA and NI lines segregate distinct mutational and transcriptional spectra [9,10], we expected contrasting networks of evolutionary coexpression, if natural selection governs network topology.

## Results and discussion

*C. elegans *microarray gene expression data were used to reconstruct evolutionary gene coexpression networks connecting genes with expression levels that covary across lines (Methods). Evolutionary gene coexpression networks were generated independently, and then compared, for the MA lines versus the NI populations. To generate evolutionary gene coexpression networks, genes are represented by nodes and the nodes (genes) are connected by an edge if they are determined to be coexpressed across lines or populations (Figure 1). Pairs of genes were determined to be coexpressed if correlation of their gene expression profiles across lines yielded an *r*-value greater than, or equal to, a defined threshold value. Results based on *r *> 0.95 are presented in the body of the manuscript, and results based on a series of differing thresholds, as well as for a different coexpression metric, are presented in Additional file 1. Since expression across five lines (populations) was evaluated for the MA and NI samples, the 0.95 *r*-value cut-off value corresponds to a *P*-value of ~0.01.

**Figure 2:**
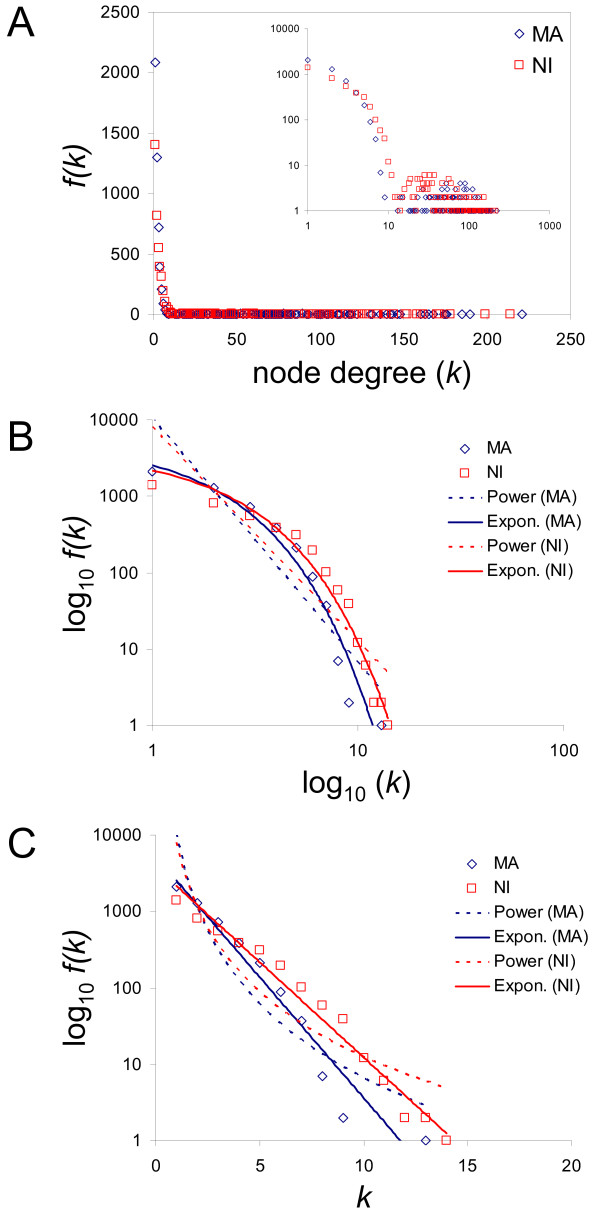
**Node degree (*k*) distributions for the MA, NI coexpression networks**. (A) The connectivity distribution [*f(k) *× *k*] is shown for the MA (blue diamonds) and NI (red squares) coexpression networks. The inset of the panel shows the same plot with the axes in log_10_-log_10 _scale. (B-C) Comparison of exponential versus power-law curve fitting to the node degree distributions, shown without the tails. The best fitting power-law (dashed lines) and exponential (solid lines) trends are shown for each distribution. (B) Node degree distributions are shown in log_10_-log_10 _scale where a power-law distribution should follow a straight line. (C) Distributions are shown in semi-log_10 _scale where an exponential distribution should follow a straight line. The data are better fit by an exponential distribution.

We first asked how node connectivity was distributed across the networks. Is connectivity distributed randomly or does it resemble the connectivities seen for a number of other complex system networks? The connectivity of any node (gene), *i.e. *the number of other genes it is linked to, is measured as its node degree (*k*). The distribution of connectivity across the MA and NI evolutionary gene coexpression networks was evaluated by observing their node degree distributions [*f(k) *× *k*]. The node degree distributions appear to be quite similar for the MA and NI coexpression networks (Figure 2A). For both MA and NI, *f(k) *decreases sharply with *k*. The vast majority of nodes have *k *≤ 10, and the distributions both have long tails, "fat" tails in the log-log plot, that correspond to nodes with anomalously high numbers of connections that are unique (or nearly so).

The node degree distributions of a number of gene coexpression networks have previously been shown to follow a power-law [4-6,12]. However, the MA and NI node degree distributions seen here are better approximated by an exponential curve. This can readily be appreciated by representing the node degree distributions as log-log (Figure 2B) and semi-log (Figure 2C) plots; power-law distributions follow a straight line on log-log plots, while exponential distributions follow a straight line on semi-log plots. Both the MA and NI distributions are better fit to a straight line on the semi-log plots. Exponential node degree distributions of this kind are more characteristic of ecological networks, such as predator-prey (food web) networks [15,16]. The exponential shape of the node degree distribution seen here indicates that the level of connectivity falls off more rapidly than seen for the other gene coexpression networks that show power-law distributions. This difference may reflect the kinds of gene expression profiles (vectors) analyzed here. We are considering changes in gene expression across lines and populations of a single species. Thus, the expression levels may not be expected to change much relative to previous coexpression studies, which have analyzed expression changes across different tissues, developmental stages, disease states and experimental conditions. Relatively uniform expression across *C. elegans *lines will not allow for the kinds of coordinated line-specific changes among genes that would lead to highly connected nodes.

**Figure 3:**
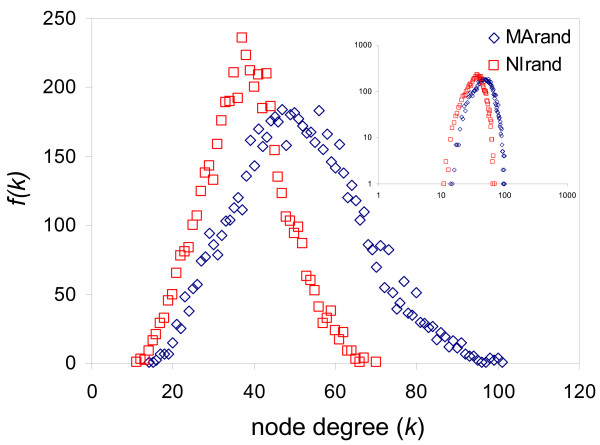
**Node degree (k) distributions for random MA and NI networks**. The connectivity distribution [*f(k) *× *k*] is shown for the random MA (blue diamonds) and random NI (red squares) coexpression networks. The inset of the figure shows the same plot with the axes in log_10_-log_10 _scale. The distributions are bell shaped and do not resemble the exponential-type distributions seen for the observed coexpression networks (Figure 1).

It is a formal possibility that the relatively low number of lines (populations), and according low dimensionality (*n *= 5) of the gene expression vectors, could result in low resolution when gene expression vectors are compared. Such a lack of resolution could lead to the artifactual appearance of similarity between MA and NI coexpression networks. We attempted to control for this possibility in three ways: i-by progressively increasing the stringency of the Pearson correlation coefficient threshold used to consider pairs of genes as coexpressed, ii-by using an independent metric for comparing gene expression vectors and iii-by building random coexpression networks using permuted gene expression datasets. When different coexpression thresholds and different vector similarity measures were used, the shapes of the node degree distributions did not change appreciably and still resemble exponential distributions with long tails [see Additional file 1 – Supplemental Figure 1]. On the other hand, generating random networks from permuted MA and NI gene expression data sets (Methods) yielded coexpression networks with radically different topologies (Figure 3 and Table 1). The MA and NI random networks have bell shaped node degree distributions with a narrower range of connectivity and resemble each other more closely than they do the observed MA and NI networks. This holds when different thresholds and different methods are used to generate random networks [see Additional file 1 – Supplemental Figure 1]. Taken together, the results of the control analyses indicate that the similar exponential-type node degree distributions observed for the MA and NI lines can not be attributed to a lack of resolution in the expression vector comparison methods. Finally, it is worth noting that our networks of coexpressed genes populate functionally related coexpression mounts as expected [9], indicating that we are not simply observing random noise in expression variation.

**Table 1:** Gene coexpression network topology parameter values and comparisons.

**Network**	**Network topology parameter values^1^**									
	***nodes***	***edges***	**<*k*>**	***<C>***	***<l>***	***<e>***	***<b>***			
***MA***	4974	10400	4.18 ± 14.24	0.25 ± 0.36	10.77 ± 5.03	20.76 ± 3.06	23,500 ± 145,912			
***NI***	4050	10790	5.33 ± 15.28	0.32 ± 0.37	10.17 ± 5.14	21.48 ± 4.22	17,440 ± 74,032			
***MA random***	7055	181001	51.31 ± 15.21	0.48 ± 0.04	6.16 ± 2.07	11.01 ± 0.12	18,203 ± 7,370			
***NI random***	5351	101215	37.83 ± 9.99	0.48 ± 0.05	6.29 ± 2.11	11.36 ± 0.51	14,140 ± 6,150			
										
**Comparison**	**Observed versus random network topology parameter values^2^**									
	**<*k*>**		***<C>***		***<l>***		***<e>***		***<b>***	
	***t***	***P***	***t***	***P***	***t***	***P***	***t***	***P***	***t***	***P***
***MA × NI***	0.45	0.65	1.11	0.27	0.68	0.50	1.12	0.26	0.31	0.76
***MA × MA random***	19.86	2.02E-86	5.34	9.58E-08	7.20	6.30E-13	26.74	4.45E-153	0.30	0.76
***NI × NI random***	14.49	4.72E-47	3.42	6.23E-04	5.62	1.92E-08	19.01	4.22E-79	0.35	0.72

^1^Parameter values are shown for the MA, NI and their corresponding random networks. For each network, the number of nodes (genes) and edges is shown along with average ± standard deviations of the node degree <*k*>, clustering coefficient *<C>*, path length *<l>*, eccentricity *<e> *and betweeness *<b>*. ^2^Average coexpression network parameter values were compared using the Students' ttest; resulting t- and *P*-values are shown.

The similarity in the connectivity distributions between the two networks was unexpected given the distinct roles of natural selection in shaping gene expression divergence among the MA versus NI populations. Apparently, the removal of effective natural selection in the MA lines does not appreciably reshape the distribution of connectivity across *C. elegans *coexpression networks. In other words, different regimes of selection do not necessarily yield different global network topological properties. To further evaluate the role of selection in shaping global topological properties, values for a number of network topology parameters were computed and compared for the MA versus NI networks (Table 1). As with the node degree distributions, the network parameters are far more similar for MA and NI than between the observed and random networks. The MA and NI networks have similar numbers of nodes and edges, while the corresponding random networks have substantially more nodes and an order of magnitude increase in the number of edges. In addition, the average node degree (<*k*>) is 4.18 and 5.33 for the MA and NI networks respectively, while *<k> *= 51.30 and 37.84 for the random networks built from the MA and NI expression data (Table 1). Taking into account their standard deviations and the number of nodes considered, the *<k> *values are statistically indistinguishable for the observed MA and NI networks, while each observed network is significantly different from its corresponding random network (Table 1). The same trend holds for the average clustering coefficients, path lengths and eccentricities; observed networks have average values that are not significantly different from one another but are highly different from the random networks. Betweeness is the only parameter that does not statistically discriminate between the observed versus random networks. As was the case with the node degree distributions, the similarity between observed network topology parameters and their differences from random networks holds when different coexpression thresholds and a different gene expression vector comparison method is used [see Additional file 1 – Supplemental Table 1].

**Figure 4:**
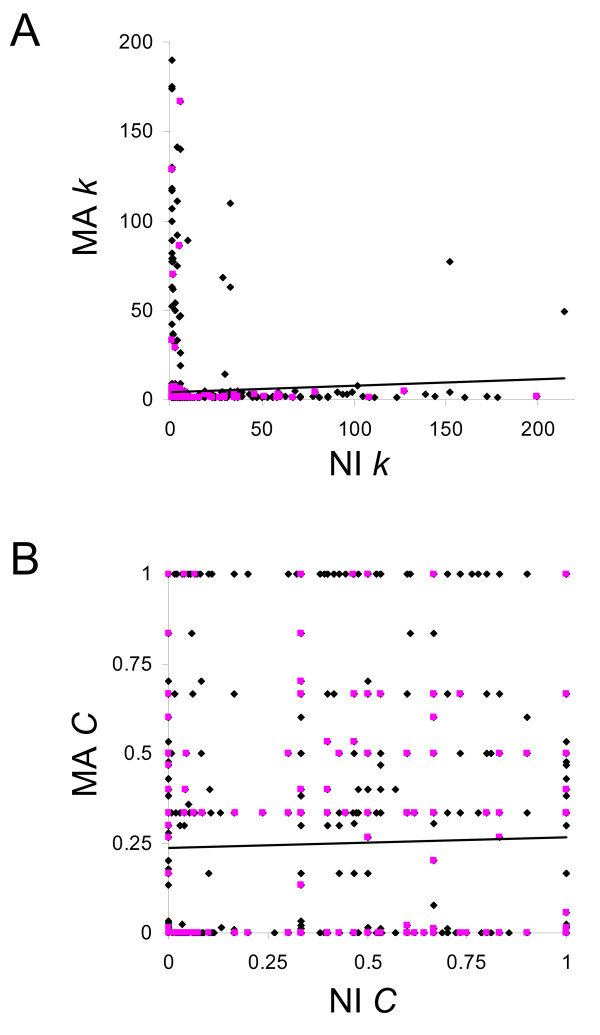
**Comparison of *k*- and *C*-values for nodes (genes) found in both the MA and NI coexpression networks**. MA- and NI-specific ordered vectors were populated with values of *k *and *C *for the 1,906 genes found in both networks. Values of *k *(A) and *C *(B) are plotted [MA × NI] and the linear trend in the data is shown. Essential genes are shown in pink and non-essential genes are shown in black.

Since the MA and NI coexpression networks were built starting from the same set of *C. elegans *genes (*i.e. *the same microarray platforms), the local connectivity properties can be directly compared by determining the fraction of edges that connect the same genes in both networks. Surprisingly, there are only seven edges, or pairs of coexpressed genes, that are shared between the MA and NI networks. This figure corresponds to a negligible 0.07% (MA) and 0.06% (NI) of the total number of edges in the two coexpression networks. The connectivity and clustering properties of the 1,906 individual nodes (genes) that are found in both the MA and NI networks were also directly compared by correlating network-specific ordered *k*- and *C*-vectors (Figure 4). These vectors have the same genes at every position with values that correspond to the *k*- and *C*-values of the genes in the MA and NI networks respectively. There is no significant positive correlation for the *k*- or *C*-values of individual nodes found in both networks (*k*: *r *= 0.04, *C*: *r *= 0.03). Thus, highly connected or clustered nodes in one network do not correspond to similarly connected or clustered nodes in the other.

Essential genes should be subject to the effects of selection in both the MA and NI lines, since individuals with lethal or sterile mutations will not be able to reproduce in any setting. Consistent with this expectation, a higher fraction of essential genes are found preserved between both networks than in either the MA or NI networks alone [see Additional file 1 – Supplemental Figure 2]. In other words, non-essential genes are freer to change between networks as the selective conditions change. Nevertheless, the lack of correlation for node topological properties between networks holds for both essential and non-essential genes (Figure 4). Thus, the essential genes that are preserved in the MA and NI networks do not have similar topological properties across networks, and so can not be responsible for the conservation of global topological properties between the networks.

As previously described for the global network properties, the relatively small number of lines (populations), and according low dimensionality (*n *= 5) of the gene expression vectors, could result in low resolution when expression vectors are compared to build coexpression networks. Therefore, the low overlap of edges between MA and NI networks (0.65%) could be due to poor sampling. In order to control for this possibility, a null distribution of *C. elegans *coexpression network overlaps was computed by randomly sampling 100 pairs of expression sets of size *n *= 5 from the Kim *et al. C. elegans *gene expression dataset [17] and computing the overlaps between coexpression networks built from these random pairs of sets [see Additional file 1 – Supplemental Figure 3]. There is an average of 2.66% conserved edges between pairs of coexpression networks built with the Kim *et al. *data with *n *= 5. This relatively low figure probably reflects that fact that different pairs of genes are coexpressed in different sets of conditions. Nevertheless, the 2.66% overlap seen for the random pairs is ~4.1× greater than the 0.65% conserved edges we observe between the MA and NI networks, and the overlap between MA and NI networks is substantially lower than any of the 100 overlaps between the network pairs from the Kim *et al. *data [see Additional file 1 – Supplemental Figure 3]. Accordingly, the difference between the observed overlap between the MA and NI networks, and the overlap between the Kim *et al. *network pairs is highly statistically significant (z = 40.7 *P *≈ 0). In other words, the low overlap between the MA and NI networks can not be explained by the size of the expression sets (*n *= 5) used in their construction.

Despite the fact that the distributions of connectivity over the entire MA and NI networks are quite similar, and very different from random, the local connections as well as the specific topological properties of the genes in the different network contexts are almost entirely different. Thus, the action of natural selection, or more appropriately the effect of removing selection, is revealed by differences in the local, but not the global, structure of the coexpression networks. This is analogous to the distinction between the conservation of global network topological properties and the divergence of local connections previously observed for orthologous human and mouse coexpression networks [6], but the pattern seen here is even more extreme. The change of specific pairs of coexpression relationships in the MA network is also consistent with previous results that showed selective constraint on gene expression divergence among NI populations and accelerated expression divergence for MA lines [9].

We sought to further evaluate the nature of the differences between the MA and NI evolutionary coexpression networks and to compare these evolutionary coexpression networks to more commonly analyzed interaction networks that link functionally associated genes. To do this, we compared pairs of evolutionarily coexpressed genes for the MA and NI networks to pairs of *C. elegans *genes previously determined to be coexpressed across 553 microarray experiments by Kim *et al. *[17]. In the study of Kim *et al.*, similarities between gene expression profiles were calculated using the Pearson correlation coefficient, and gene expression profile similarities (distances) were converted into two dimensions using force-directed placement. This procedure resulted in a *C. elegans *gene expression topological map where the proximity of pairs of genes in two dimensions (*x*, *y*) corresponds to their degree of coexpression across conditions, and presumably their functional relatedness. We evaluated the way that MA versus NI coexpressed gene pairs, along with the random gene pairs for each network, populate the *C. elegans *gene expression topological map by measuring Euclidean distances on the topological map between pairs of genes in the MA, NI or random networks. Coexpressed gene pairs in both the MA and NI networks are significantly more closely grouped on the *C. elegans *topological map than are pairs of genes from the corresponding random networks (2.5<*t*<23.4 3.6e-49<*P *< 1.1e-2 Student's ttest). This indicates that functionally relevant interactions are captured by both the MA and NI networks. Interestingly, MA coexpressed gene pairs are more closely grouped, on average, than NI coexpressed gene pairs on the *C. elegans *gene expression topological map (*t *= 14.78 *P *= 3.6e-49 Student's ttest). This result is similar to that reported by Denver and colleagues [9] who showed that genes differentially expressed in MA lines tended to cluster in specific coexpression "mounts," and concluded that this was likely caused by trans- acting mutations purged by selection from NI lines. Changes in expression across conditions recorded on the *C. elegans *topological map are due largely to the context-dependent action of transcription factors. Accordingly, the local differences between the MA and NI evolutionary coexpression networks are indicative of network rewiring, likely caused by mutations in trans- acting factors that 'capture' gene expression modules in the MA lines. Thus, the action of natural selection, or more appropriately the effect of removing selection, is only revealed by differences in the local structure of the coexpression networks.

## Conclusion

The appearance of similar global topological properties across disparate complex biological systems led to the view that there were 'universal laws' that governed the function and evolution of cellular networks [7,18]. It followed that the revelation of such laws, via the analytical tools of network theory, could yield revolutionary insight into biology. However, some reserved judgment as to whether such universal laws existed and if the statistical analysis of network topologies would reveal something non-trivial about biological systems [19]. This initial agnosticism as to the ability of the network approach to reveal fundamental and novel biological principles has hardened into a deep skepticism regarding its very relevance [8]. The pessimistic view of the network approach to biology is based in large part on the assertion that similar topological properties do not entail similar network architectures or functional constraints, and analogous conclusions have been reached for computer networks [20]. Indeed, it has been shown that similar global network topological properties can emerge due to non-adaptive processes, such as simple birth-and-death models [21,22], without any assumption of selection [23]. Our own comparison of the MA versus NI evolutionary gene coexpression networks has revealed that similar properties at a high level of abstraction can obscure substantial and biologically relevant differences at lower levels. With respect to the evolution of biological systems, the details remain important.

## Methods

### Gene expression data

*C. elegans *gene expression data from [9] were downloaded from the Stanford Microarray Database . We analyzed gene expression data for 7,056 genes across four MA lines (MA24, MA41, MA83 and MA99) and their common ancestor laboratory strain (N2) along with 5,350 genes across five NI populations (AB1, CB4856, N2, PB303, PB306 and N2). Gene expression levels were originally determined using cDNA microarrays on developmentally synchronized populations with a loop experimental design as previously described [9]. For each line or population (*i.e. *each individual microarray experiment), all gene expression levels (hybridization intensities) *g*_*i *_were *z*-normalized by subtracting the mean intensity over all genes for the experiment from the individual intensity and dividing by the standard deviation (*sd*) of gene intensities for the experiment:

$$zg_{i}=(g_{i}-\bar{g_{i}})/sd(g_{i})$$

### Gene coexpression networks

Coexpression networks were reconstructed independently for MA lines and NI populations. In the gene coexpression networks, gene as taken as nodes and pairs of nodes (genes) are connected by an edge if they are coexpressed across lines (populations). To determine if pairs of genes are coexpressed, the expression profile for each gene is taken as its normalized expression levels across MA or NI lines (populations). Thus, each gene *g*_*i *_was represented as a row vector with dimensions (*n *= 5) equal to the number of MA lines or NI populations evaluated:

*g*_*i *_= [*zg*_*i*1_, *zg*_*i*2 _... *zg*_*in*_]

With *m *genes considered, the expression data for all genes across all lines or populations are represented as an *m *× *n *matrices for MA and NI. All pairs (*x*, *y*) of gene expression vectors (profiles) from the MA and NI *m *× *n *matrices were compared using the Pearson correlation coefficient (*r*) and the Euclidean distance (*ed*):

$$r=\frac{\sum (x-\bar{x})(y-\bar{y})}{{\sum \left(x-\bar{x}\right)}^{2}\sum {\left(y-\bar{y}\right)}^{2}}$$

$$ed=\sqrt{{\left(x_{1}-y_{1}\right)}^{2}+{\left(x_{2}-y_{2}\right)}^{2}+...{\left(x_{n}-y_{n}\right)}^{2}}$$

Pairs of genes that have values *r *or *ed *that exceeded a given threshold were considered to be coexpressed and were thus represented as nodes connected by an edge in the MA or NI coexpression network. For the Pearson correlation coefficient, *r*-value thresholds used were 0.95, 0.96, 0.97, 0.98 and 0.99. Five Euclidean distance value thresholds were computed so that the resulting networks would have the same number of edges as the Pearson correlation coefficient networks: *ed *= 0.1942, 0.1855, 0.1744, 0.1586, and 0.1381.

The Pearson correlation coefficient and Euclidean distance are widely used in the analysis of gene expression data. The choice of these two metrics was also based in part on previous results that showed they were the most dissimilar of the distance metrics commonly used to compare and cluster expression data [6]. In this sense, they represent a conservative pair of measures to be used when controlling for methodological effects on the results.

Random pairs of gene coexpression networks were built using the *C. elegans *gene expression data from the study of Kim *et al. *[17]. The Kim *et al. *data set includes 553 microarray experiments over a variety of experimental conditions, including different growth conditions, developmental stages and mutants. For 1,000 genes, 2 sets of 5 experiments each were randomly sampled from the 553 experiments in the *C. elegans *gene expression data set. Each paired set of experiments, and the resulting sets gene expression vectors, was used as described above to construct pairs of coexpression networks. This procedure was repeated 100 times, and for each replicate pair, the overlap between the networks, in percentage of conserved edges, was computed.

### Statistical analyses of network topologies

The number of nodes and edges for each coexpression network along with the average and standard deviation values for the following network parameters – node degree (*k*), clustering coefficient (*C*), path length (*l*), eccentricity (*e*) and betweeness (*b*) – were calculate using the program tYNA [24]. The node degree (*k*) is simply the number of connections (edges) for any given node. The clustering coefficient (*C*) is a measure of how connected the neighbors of a given node are:

*C *= 2*n*/*k*(*k *- 1)

where *n *is the number of links among the *k *neighbors of the node. Path length (*l*) is the shortest path (geodesic), in terms of numbers of edges that are traversed, between any two nodes in the connected part of the network. The eccentricity (*e*) of a node is the largest geodesic between that node and any other node in the network. The betweeness (*b*) of a node is the number of geodesics that pass through the node. Average network parameter values were compared between networks using the Students' ttest. Node degree distributions were computed as f(*k*) × *k*, and curve fitting on the distributions was done using least squares.

Random networks were generated by randomly shuffling the positions of the expression values within the original MA and NI *m *× *n *data matrices. The shuffled MA and NI datasets were used to re-calculate all pairwise correlation (*r*) or Euclidean distance (*ed*) values between gene expression profiles (vectors), and the same threshold cutoffs as employed for the observed networks were used to make coexpression networks from the random *r*- or *ed*-values.

### Comparison with the *C. elegans *gene expression topological map

In [17], *C. elegans *gene expression data for were taken from 553 microarray experiments over a variety of experimental conditions, including different growth conditions, developmental stages and mutants. For all pairs of *C. elegans *genes analyzed, distances between profiles of expression levels were calculated using the Pearson correlation coefficient, and gene expression profile distances were converted into two dimensions using force-directed placement [17]. This procedure results in the *C. elegans *gene expression topological map where the proximity of pairs of genes in two dimensions (*x*, *y*) corresponds to their degree of coexpression across conditions. The topological map (*x*, *y*) coordinates for all genes analyzed were taken from the supplemental data website . For pairs of coexpressed genes that are linked in the MA and NI networks, the *C. elegans *gene expression topological distance was computed by taking the Euclidean distance (*Ed*) for each pair of corresponding (*x*, *y*) coordinates. *E.g. *for gene1 (*g1*) versus gene2 (*g2*):

$$Ed_{12}=\sqrt{{\left(x_{1}-x_{2}\right)}^{2}+{\left(y_{1}-y_{2}\right)}^{2}}$$

Euclidean distances were then averaged over all pairs in each network and then compared for the MA and NI networks using the Student's ttest.

## Abbreviations

MA: mutation accumulation; NI: natural isolate

## Authors' contributions

IKJ, DRD and JTS conceived of the study, and participated in its design and coordination and helped to draft the manuscript. LSK and IKJ performed the computational analysis of the evolutionary gene coexpression networks. All authors read and approved the final manuscript.

## Supplementary Material

Additional file 1 **Supplemental Information.** Supplemental Figures 1 – 3 and Supplemental Table 1. Click here for file

## References

[B1] Gu Z, Nicolae D, Lu HH, Li WH (2002). Rapid divergence in expression between duplicate genes inferred from microarray data. Trends Genet.

[B2] Luscombe NM, Babu MM, Yu H, Snyder M, Teichmann SA, Gerstein M (2004). Genomic analysis of regulatory network dynamics reveals large topological changes. Nature.

[B3] Makova KD, Li WH (2003). Divergence in the spatial pattern of gene expression between human duplicate genes. Genome Res.

[B4] Agrawal H (2002). Extreme self-organization in networks constructed from gene expression data. Phys Rev Lett.

[B5] Bergmann S, Ihmels J, Barkai N (2004). Similarities and differences in genome-wide expression data of six organisms. PLoS Biol.

[B6] Tsaparas P, Marino-Ramirez L, Bodenreider O, Koonin EV, Jordan IK (2006). Global similarity and local divergence in human and mouse gene co-expression networks. BMC Evol Biol.

[B7] Barabasi AL, Oltvai ZN (2004). Network biology: understanding the cell's functional organization. Nat Rev Genet.

[B8] Keller EF (2005). Revisiting "scale-free" networks. Bioessays.

[B9] Denver DR, Morris K, Streelman JT, Kim SK, Lynch M, Thomas WK (2005). The transcriptional consequences of mutation and natural selection in Caenorhabditis elegans. Nat Genet.

[B10] Vassilieva LL, Hook AM, Lynch M (2000). The fitness effects of spontaneous mutations in Caenorhabditis elegans. Evolution.

[B11] Jordan IK, Marino-Ramirez L, Koonin EV (2005). Evolutionary significance of gene expression divergence. Gene.

[B12] Jordan IK, Marino-Ramirez L, Wolf YI, Koonin EV (2004). Conservation and coevolution in the scale-free human gene coexpression network. Mol Biol Evol.

[B13] Liao BY, Zhang J (2006). Evolutionary conservation of expression profiles between human and mouse orthologous genes. Mol Biol Evol.

[B14] Rifkin SA, Houle D, Kim J, White KP (2005). A mutation accumulation assay reveals a broad capacity for rapid evolution of gene expression. Nature.

[B15] Dunne JA, Williams RJ, Martinez ND (2002). Network structure and biodiversity loss in food webs: robustness increases with connectance. Ecology Letters.

[B16] Montoya JM, Pimm SL, Sole RV (2006). Ecological networks and their fragility. Nature.

[B17] Kim SK, Lund J, Kiraly M, Duke K, Jiang M, Stuart JM, Eizinger A, Wylie BN, Davidson GS (2001). A gene expression map for Caenorhabditis elegans. Science.

[B18] Barabasi AL (2002). Linked: the new science of networks.

[B19] Wolf YI, Karev G, Koonin EV (2002). Scale-free networks in biology: new insights into the fundamentals of evolution?. Bioessays.

[B20] Lun L, David A, Walter W, John D (2004). A first-principles approach to understanding the internet's router-level topology. Proceedings of the 2004 conference on Applications, technologies, architectures, and protocols for computer communications.

[B21] Karev GP, Wolf YI, Rzhetsky AY, Berezovskaya FS, Koonin EV (2002). Birth and death of protein domains: a simple model of evolution explains power law behavior. BMC Evol Biol.

[B22] Koonin EV, Wolf YI, Karev GP (2002). The structure of the protein universe and genome evolution. Nature.

[B23] Lynch M (2007). The evolution of genetic networks by non-adaptive processes. Nat Rev Genet.

[B24] Yip KY, Yu H, Kim PM, Schultz M, Gerstein M (2006). The tYNA platform for comparative interactomics: a web tool for managing, comparing and mining multiple networks. Bioinformatics.

