# Supplementary material for: Natural selection governs local, but not global, evolutionary gene coexpression networks in Caenorhabditis elegans
Source: BMC Syst Biol. 2008 Nov 13;2:96. doi: 10.1186/1752-0509-2-96 (PMC2596099; doi:10.1186/1752-0509-2-96)

Supplemental Figure 1. **Node degree distribution comparisons.** Node degree (*k*) distributions for the MA, NI and corresponding random coexpression networks generated using different gene expression vector comparison methods (Pearson correlation coefficient and Euclidean distance) and different cut-off thresholds are shown. For each plot, the connectivity distribution [*f(k)* × *k*] is shown for the MA (blue diamonds), NI (red squares) and corresponding random coexpression networks. The inset of the figures shows the same plots with the axes in log10-log10 scale.

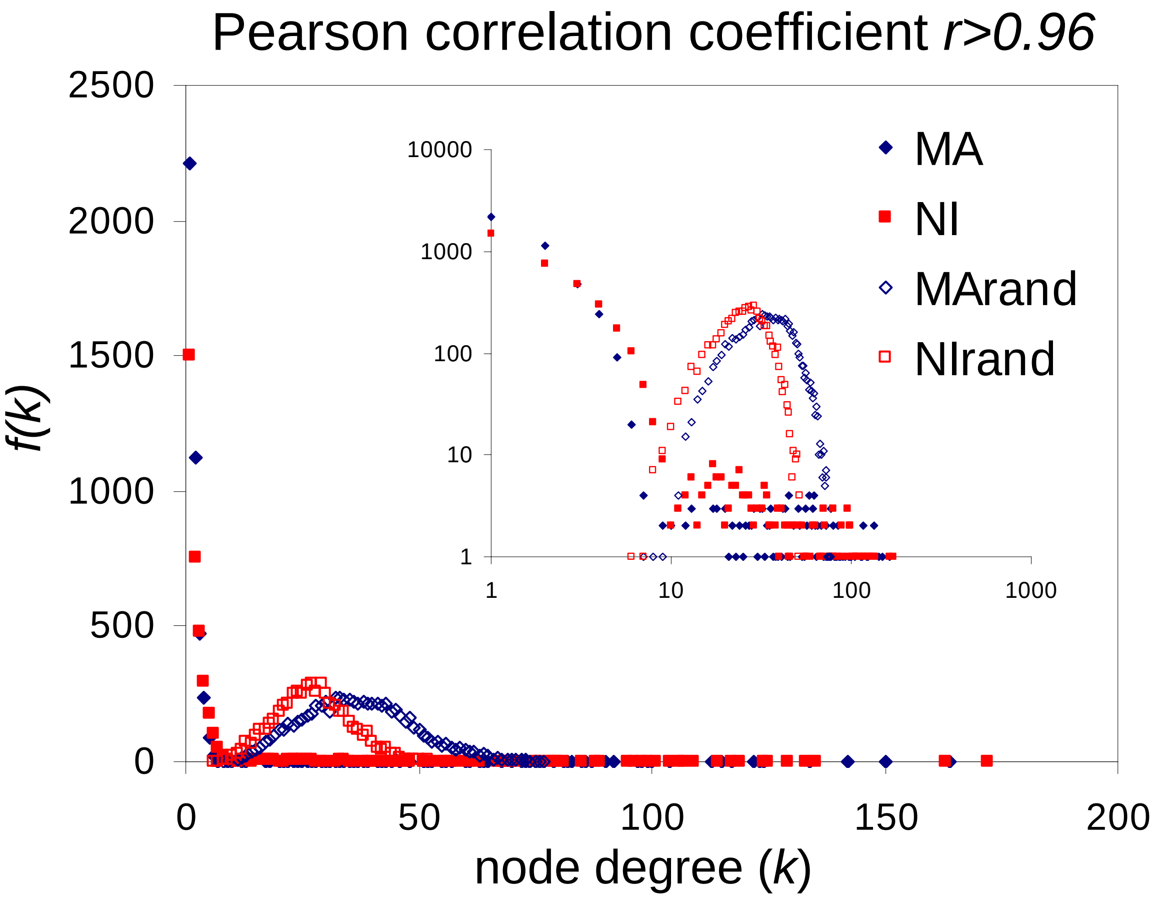

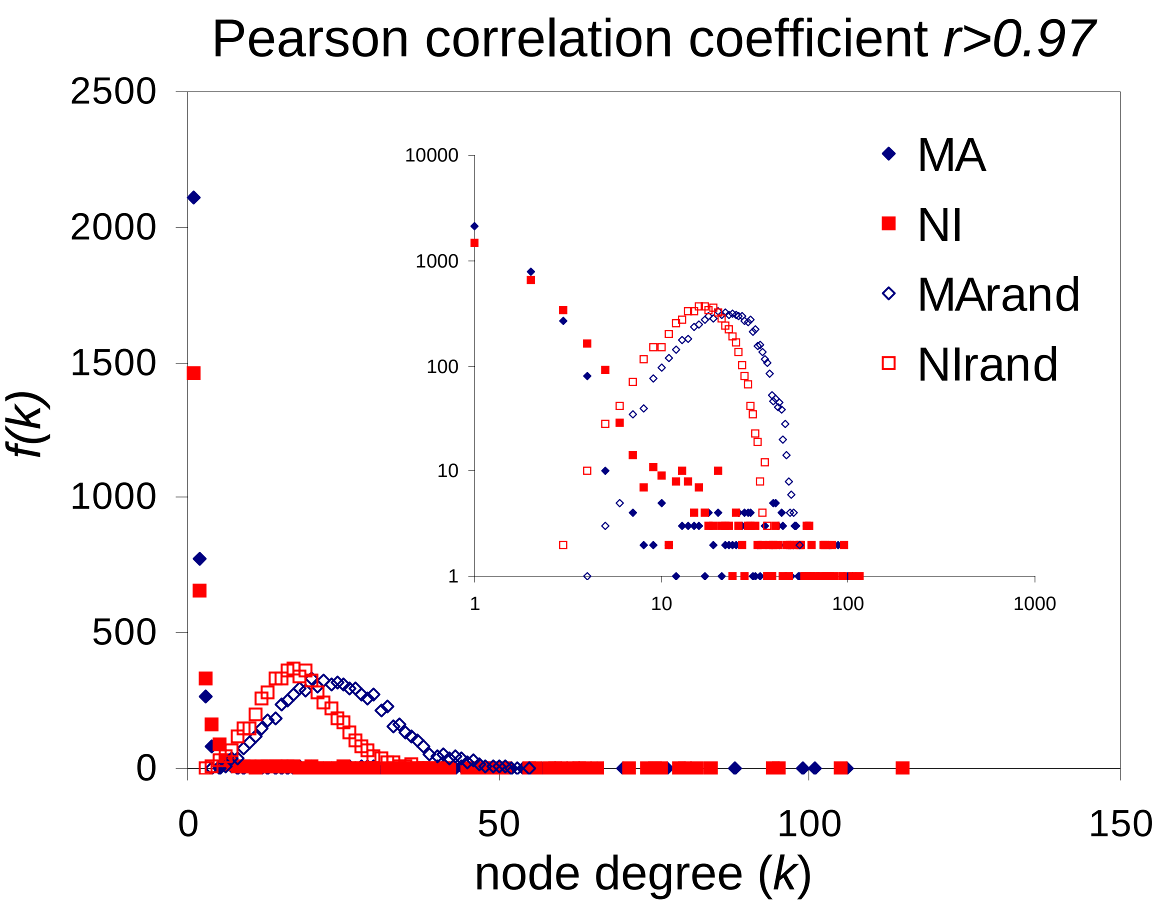

Pearson correlation coefficient r>0.97
f(k)
node degree (k)
0
500
1000
1500
2000
2500
0
50
100
150
MA
NI
MArand
NIrand
1
10
100
1000
10000
1
10
100
1000

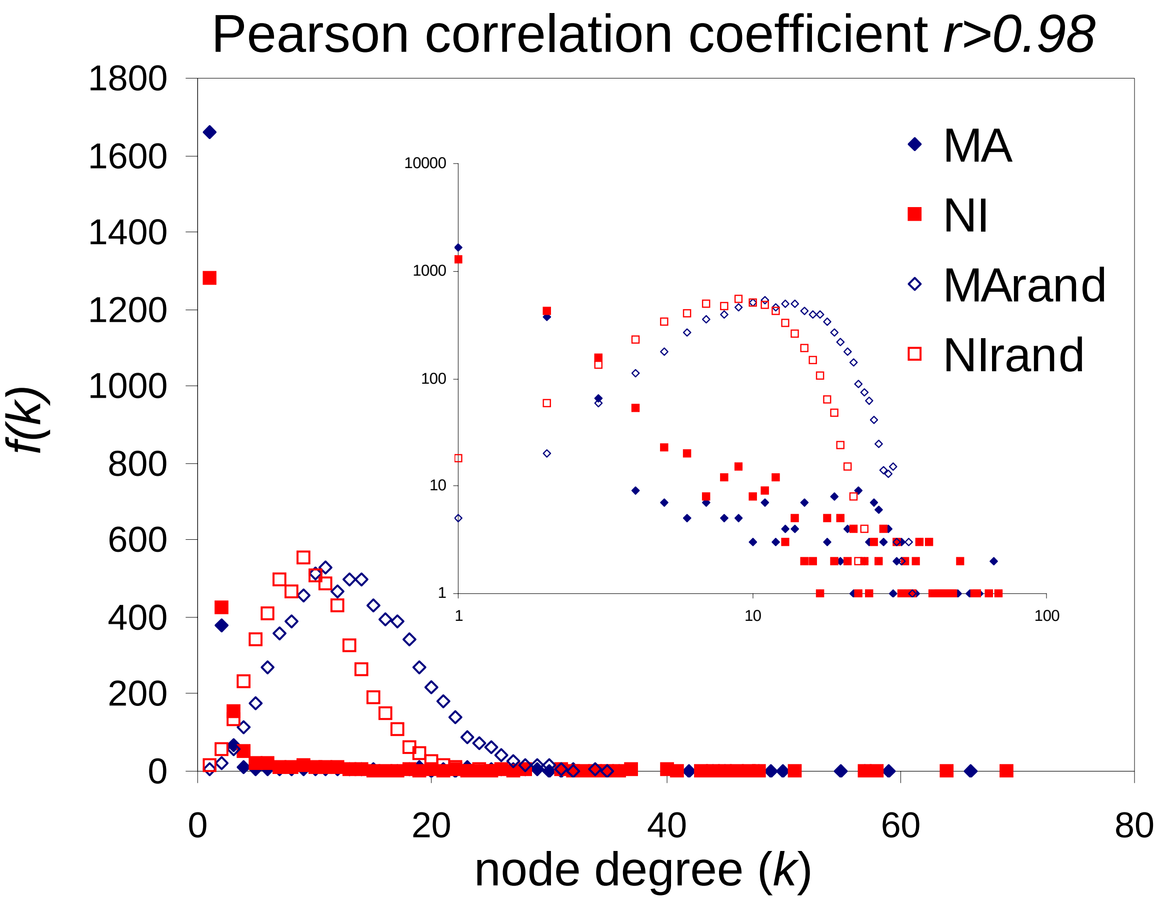

Pearson correlation coefficient *r>0.98*
f(k)
node degree (k)
MA
NI
MArand
NIrand

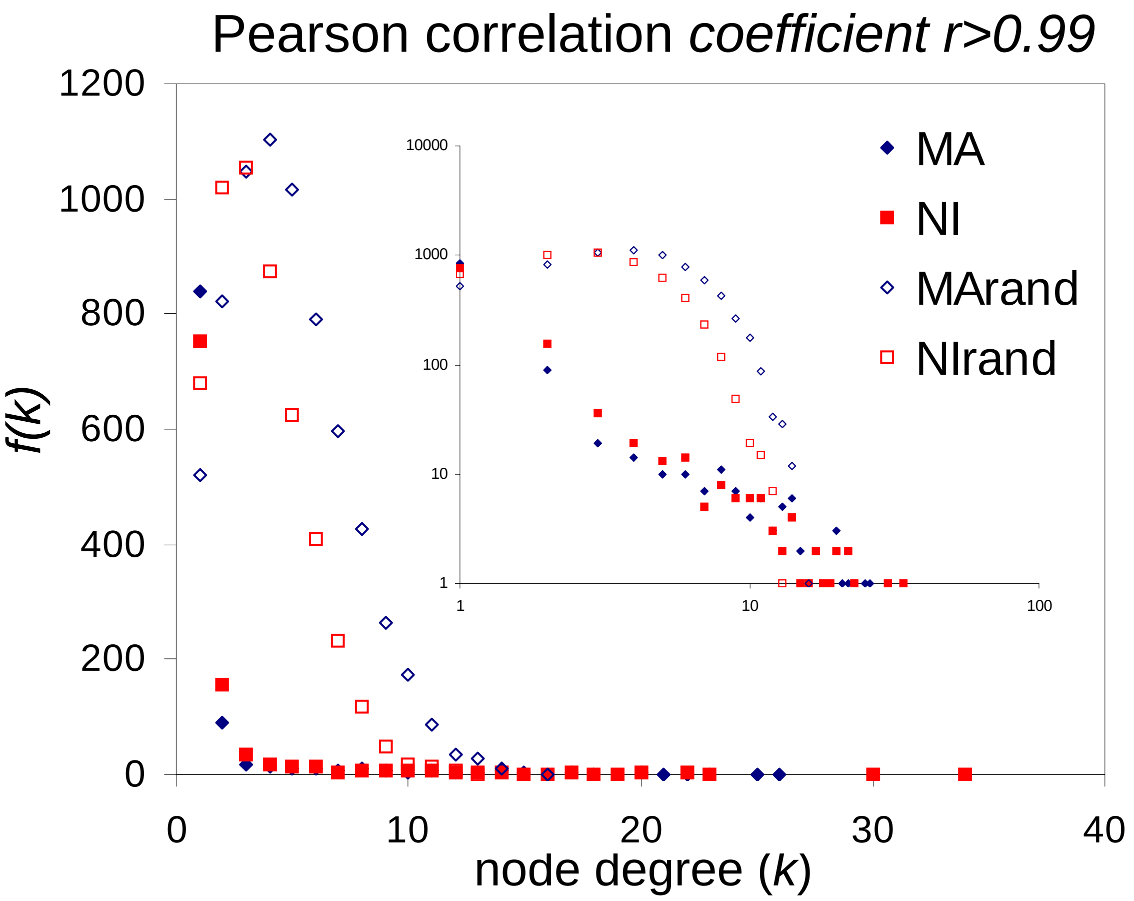

Pearson correlation *coefficient r>0.99*
f(k)
node degree (k)
MA
NI
MArand
NIrand
0
200
400
600
800
1000
1200
0
10
20
30
40
1
10
100
1000
10000
1
10
100

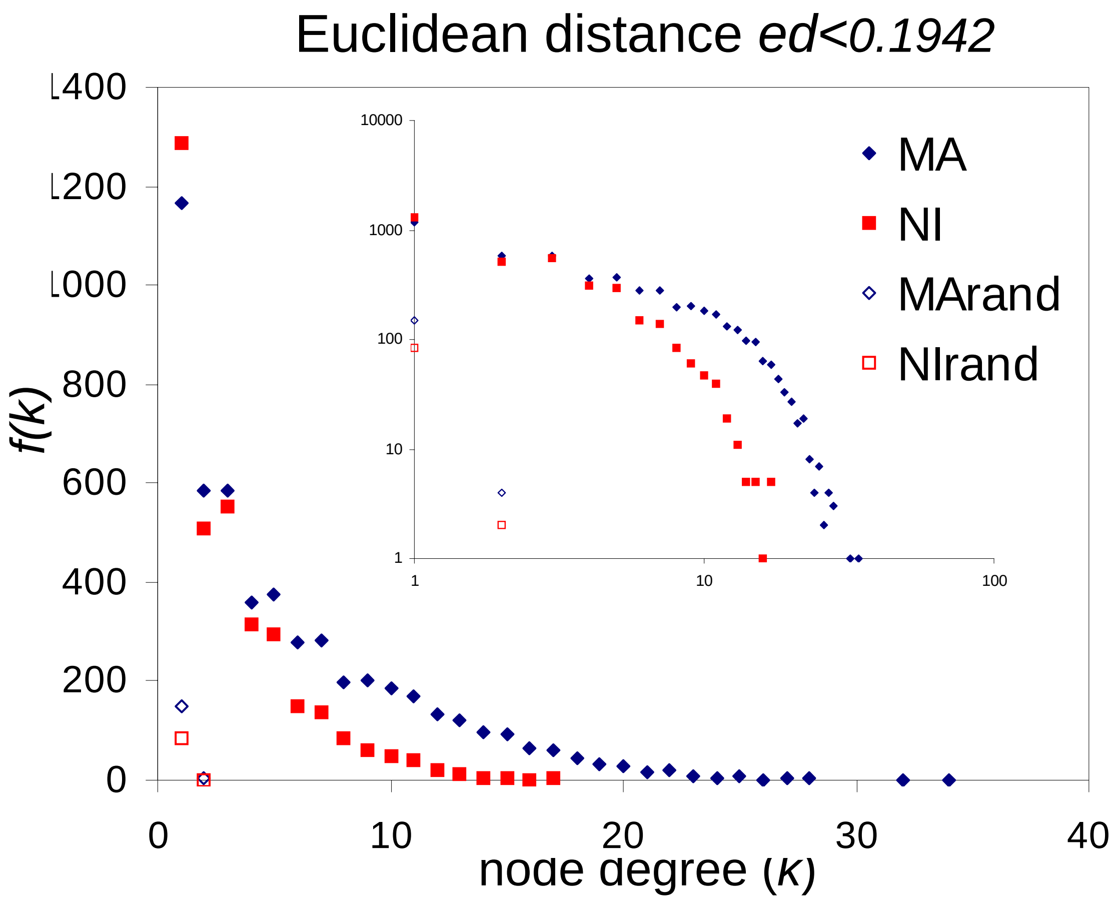

Euclidean distance ed<0.1942
f(k)
node degree (k)
MA
NI
MArand
NIrand

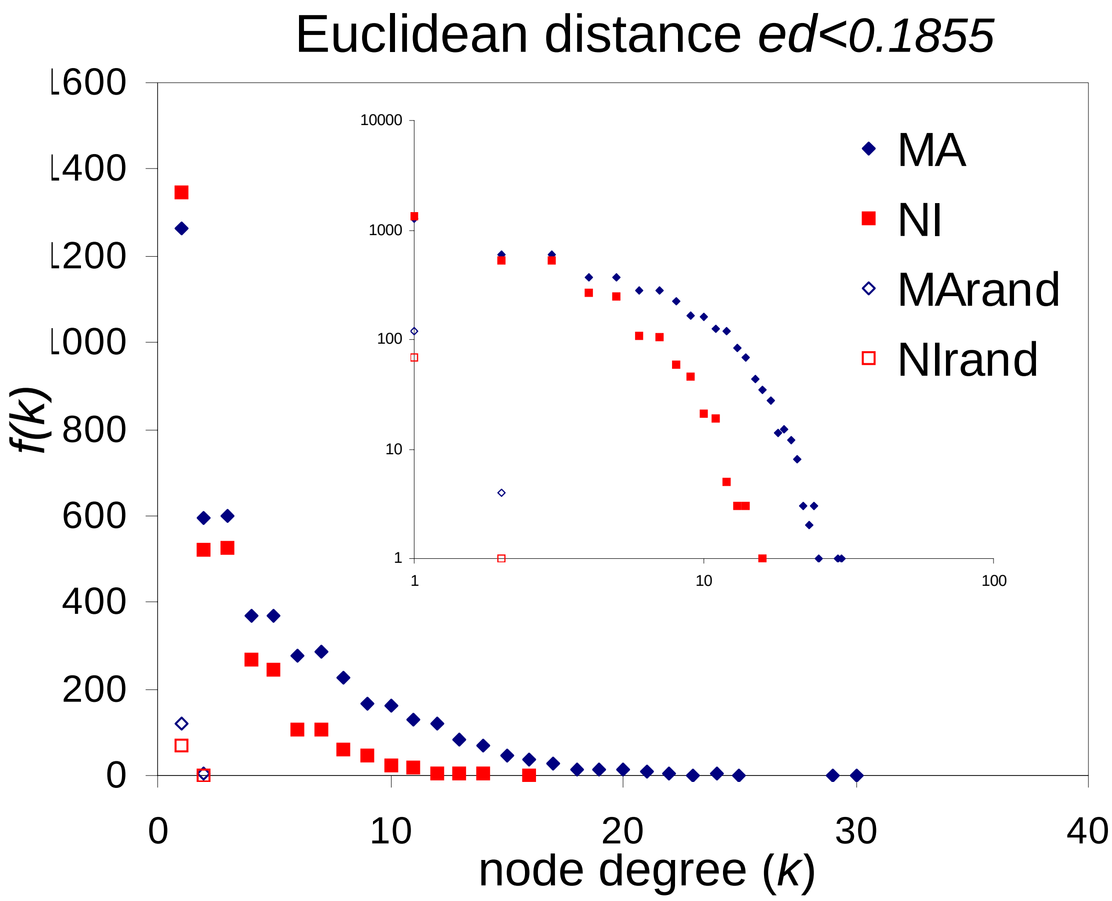

Euclidean distance ed<0.1855
f(k)
node degree (k)
MA
NI
MArand
NIrand
1600
1400
1200
1000
800
600
400
200
0
0
10
20
30
40
10000
1000
100
10
1
1
10
100

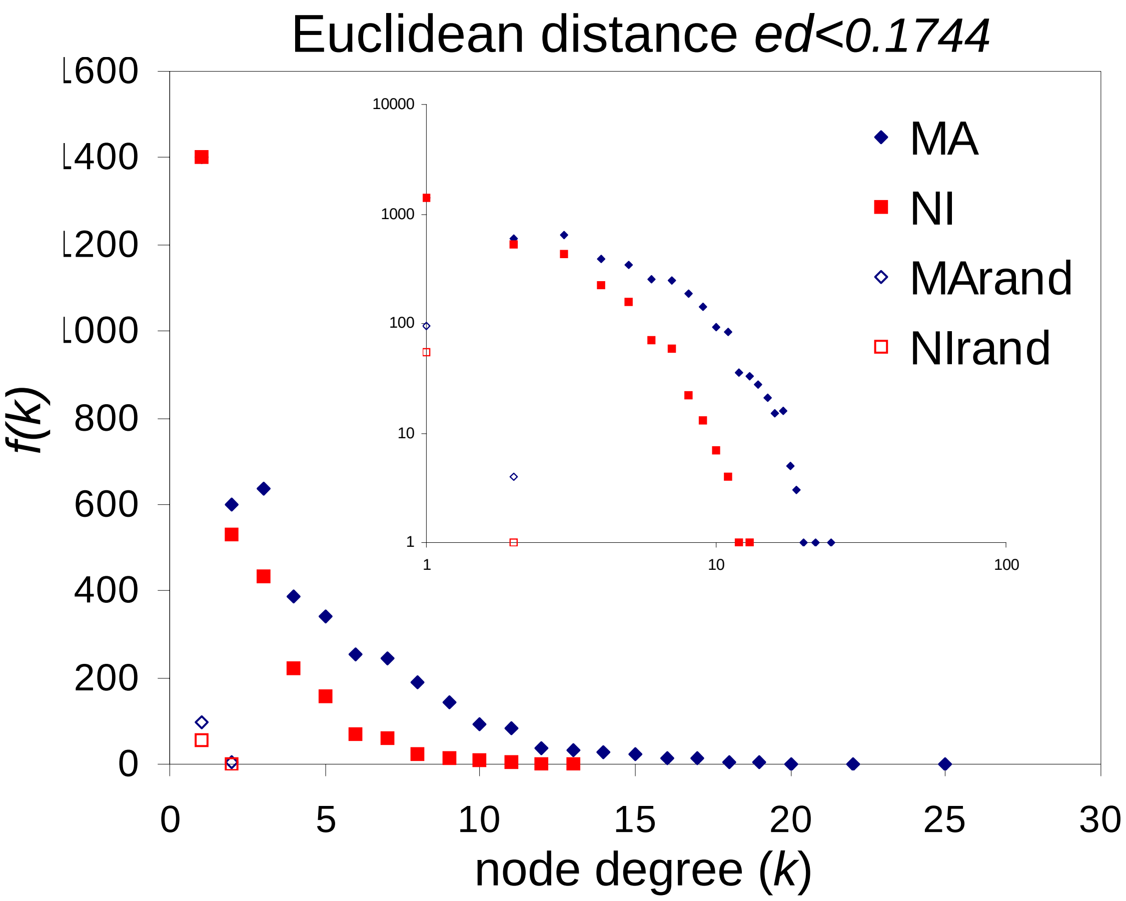

Euclidean distance ed<0.1744
f(k)
node degree (k)
MA
NI
MArand
NIrand
1600
1400
1200
1000
800
600
400
200
0
0
5
10
15
20
25
30
10000
1000
100
10
1
1
10
100

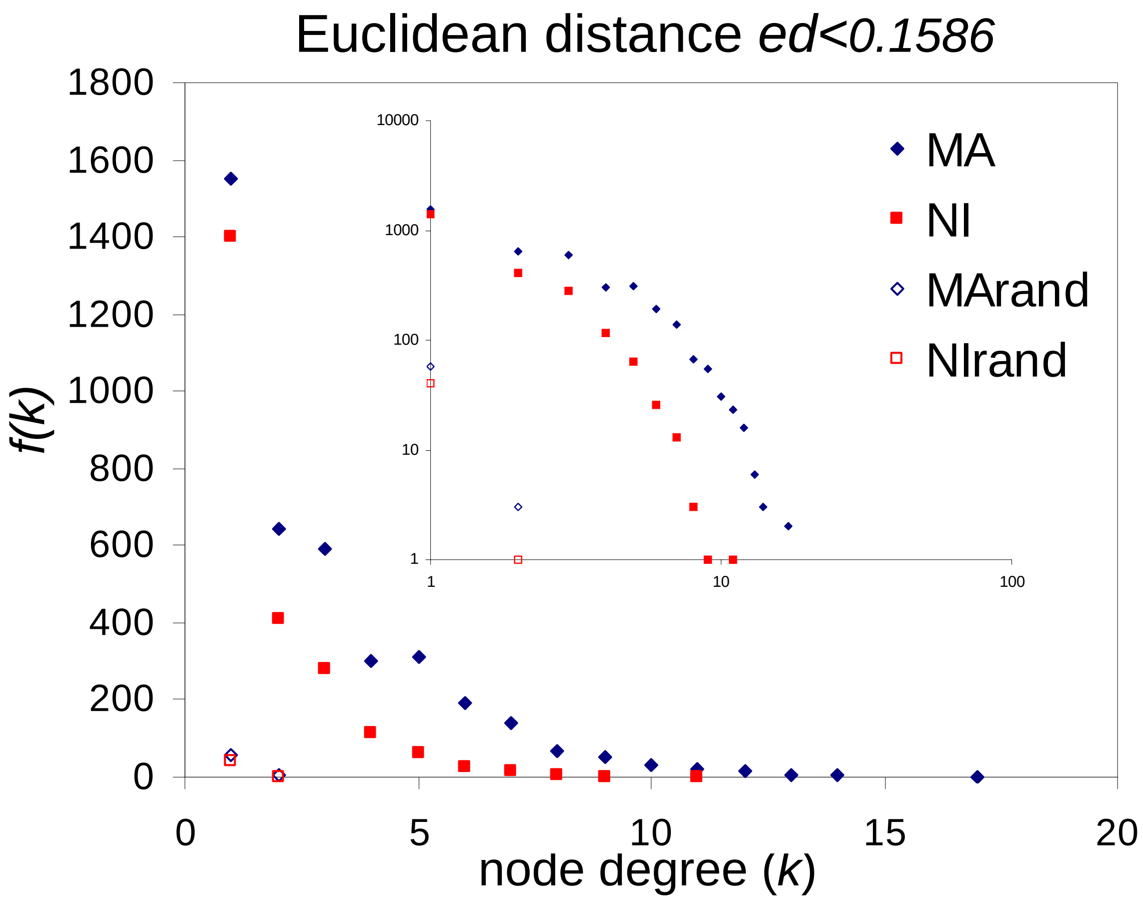

Euclidean distance ed<0.1586
f(k)
node degree (k)
MA
NI
MArand
NIrand
1800
1600
1400
1200
1000
800
600
400
200
0
0
5
10
15
20
10000
1000
100
10
1
1
10
100

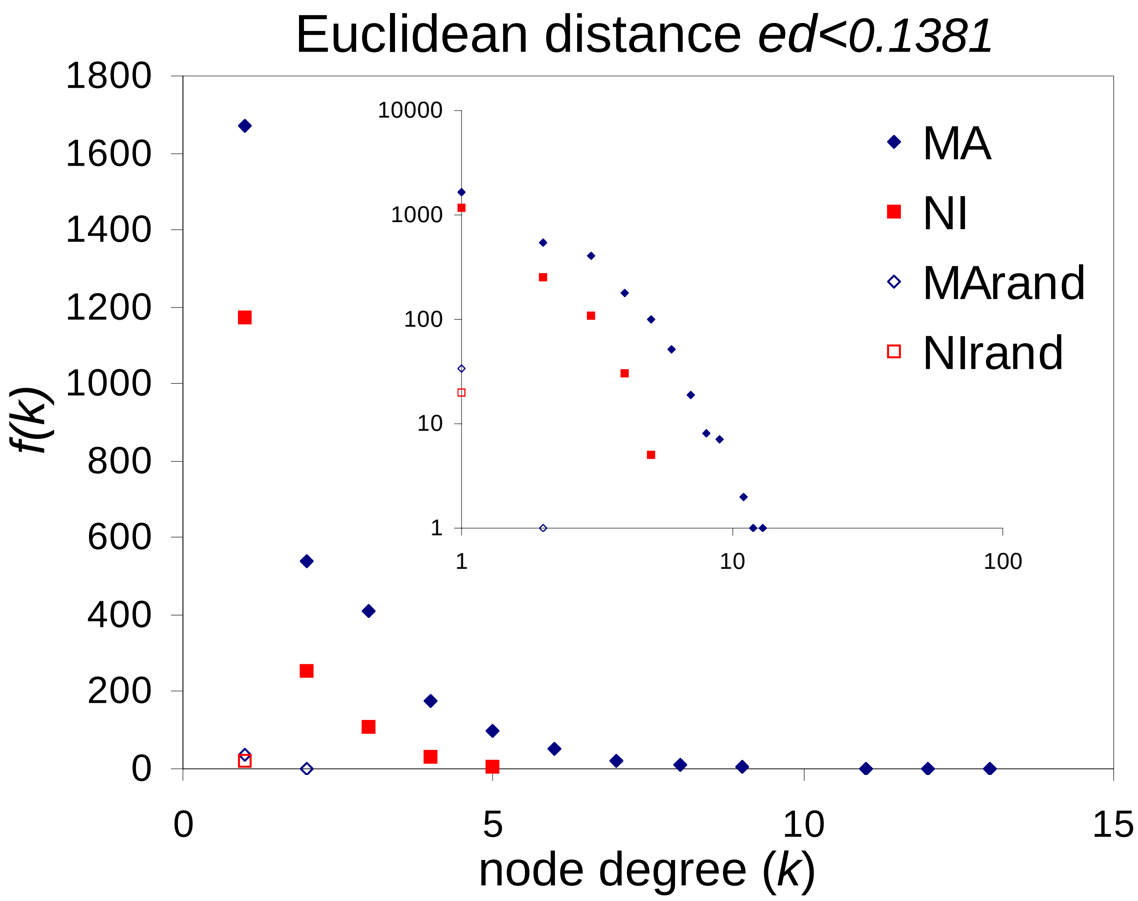
Euclidean distance ed<0.1381
f(k)
1800
1600
1400
1200
1000
800
600
400
200
0
0
5
10
15
node degree (k)
10000
1000
100
10
1
1
10
100
MA
NI
MArand
NIrand

Supplemental Table 1. **Gene coexpression network parameter values.** Gene coexpression network topology parameters are shown for networks generated using different gene expression vector comparison methods (Pearson correlation coefficient and Euclidean distance) and different cut-off thresholds. Parameter values are shown for the MA, NI and their corresponding random networks. For each network, the number of nodes (genes) and edges is shown along with average ± standard deviations of the node degree <*k*>, clustering coefficient <*C*>, path length <*l*>, eccentricity <*e*> and betweeness <*b*>. Cut-off values are described in the Methods section.

**Pearson correlation coefficient r > 0.95**							
	**nodes**	**edges**	**<*k*>**	**<*C*>**	**<*l*>**	**<*e*>**	**<*b*>**
**MA**	4974	10400	4.18 ± 14.24	0.25 ± 0.36	10.77 ± 5.03	20.76 ± 3.06	23,500 ± 145,912
**NI**	4050	10790	5.33 ± 15.28	0.32 ± 0.37	10.17 ± 5.14	21.48 ± 4.22	17,440 ± 74,032
**MA random**	7055	181001	51.31 ± 15.21	0.48 ± 0.04	6.16 ± 2.07	11.01 ± 0.12	18,203 ± 7,370
**NI random**	5351	101215	37.83 ± 9.99	0.48 ± 0.05	6.29 ± 2.11	11.36 ± 0.51	14,140 ± 6,150
**Pearson correlation coefficient r > 0.96**							
	**nodes**	**edges**	**<*k*>**	**<*C*>**	**<*l*>**	**<*e*>**	**<*b*>**
**MA**	4301	7501	3.49 ± 11.10	0.21±0.36	7.03 ± 3.01	12.50 ± 3.62	53,266.66 ± 25,964.08
**NI**	3574	7820	4.38 ± 12.00	0.28 ± 0.37	11.34 ± 5.92	24.53 ± 7.75	15,092.74 ± 70,633.94
**MA random**	7057	130245	36.91±11.72	0.48±0.05	7.03±2.37	12.99±0.20	21,253.76±9,858.76
**NI random**	5351	72773	27.20 ± 7.78	0.48 ± 0.06	7.20 ± 2.43	13 ± 0.23	16,571.25 ± 8,165.60

**Pearson correlation coefficient r > 0.97**							
	**nodes**	**edges**	**<*k*>**	**<*C*>**	**<*l*>**	**<*e*>**	**<*b*>**
**MA**	3376	4856	2.88 ± 8.13	0.14 ± 0.31	7.81 ± 3.48	12.19 ± 4.56	3,279.89 ± 16,905.96
**NI**	2928	5184	3.54 ± 8.86	0.23 ± 0.37	13.95±7.75	27.28±11.64	12,834.80±76,951
**MA random**	2928	5184	3.54±8.86	0.23±0.37	13.95±7.75	27.28±11.64	12,834.80±76,951
**NI random**	5351	47404	17.72±5.84	0.47±0.08	8.72±2.95	15.97±0.33	20,635.23
**Pearson correlation coefficient r > 0.98**							
	**nodes**	**edges**	**<*k*>**	**<*C*>**	**<*l*>**	**<*e*>**	**<*b*>**
**MA**	2245	2645	2.36±5.47	0.09±0.28	9.07±5.28	9.36±7.03	1,213.42±8,662.77
**NI**	2097	2886	2.75±5.79	0.14±0.31	10.79±7.58	17.52±12.02	2,411.19±10,941.35
**MA random**	7057	46698	13.23±5.28	0.47±0.11	11.16±3.79	20.43±0.61	35,817.76±27020.70
**NI random**	5348	25853	9.67±3.90	0.47±0.15	11.90±4.01	21.82±0.78	29,137.19±24,149.92

**Pearson correlation coefficient r > 0.99**							
	**nodes**	**edges**	**<*k*>**	**<*C*>**	**<*l*>**	**<*e*>**	**<*b*>**
**MA**	1047	997	1.90±2.92	0.04±0.18	4.22±2.73	3.08±2.23	44.04±351.72
**NI**	1047	1036	1.98±2.93	0.06±0.23	5.78±3.14	5.91±4.45	139.48±669.16
**MA random**	6926	16587	4.79±2.52	0.43±0.28	22.77±7.83	40.36±10.60	64,958.74±98,780.19
**NI random**	5104	9214	3.61±1.98	0.41±0.33	31.71±11.72	48.82±22.65	51,194.67±119,481.51
**Euclidean<0.1942**							
	**nodes**	**edges**	**<*k*>**	**<*C*>**	**<*l*>**	**<*e*>**	**<*b*>**
**MA**	5119	15221	5.95±5.04	0.31±0.28	17.73±10.25	37.59±15.63	31,637.06±130,874.26
**NI**	3525	5901	3.35±2.64	0.24±0.31	21.79±12.71	41.54±24.41	19,869.27±88,293.93
**MA random**	154	79	1.03±0.16	0.02±0.14	1.50±0.50	2.00±0.00	0.01±0.08
**NI random**	88	45	1.02±0.15	0.00±0.00	1.51±0.50	2.00±0.00	0.02±0.15

**Euclidean<0.1855**							
	**nodes**	**edges**	***<k>***	***<C>***	***<l>***	***<e>***	***<b>***
**MA**	4873	12306	5.05±4.21	0.29±0.29	18.86±10.70	38.33±17.12	30,502.82±138,523.23
**NI**	3278	4760	2.90±2.22	0.22±0.31	23.75±13.71	38.14±25.89	16,496.42±83,274.50
**MA random**	126	65	1.03±0.18	0.02±0.15	1.50±0.50	2.00±0.00	0.01±0.09
**NI random**	71	36	1.01±0.12	0.00±0.00	1.50±0.50	2.00±0.00	0.01±0.12
**Euclidean<0.1744**							
	**nodes**	**edges**	***<k>***	***<C>***	***<l>***	***<e>***	***<b>***
**MA**	4536	9287	4.09±3.34	0.27±0.30	21.44±12.38	42.39±21.78	29,274.51±126,502.50
**NI**	2927	3545	2.42±1.75	0.20±0.32	25.95±14.91	30.53±27.43	9,654.39±45,018.92
**MA random**	102	53	1.04±0.19	0.03±0.17	1.50±0.50	2.00±0.00	0.01±0.10
**NI random**	57	29	1.02±0.13	0.00±0.00	1.50±0.50	2.00±0.00	0.02±0.13

**Euclidean<0.1586**								
	**nodes**	**edges**	**<*k*>**	**<*C*>**	**<*l*>**	**<*e*>**	**<*b*>**
**MA**	3928	5978	3.04±2.35	0.23±0.31	26.52±15.70	41.49±28.49	22,265.94±92,543.52
**NI**	2314	2193	1.90±1.26	0.16±0.32	19.80±11.32	11.72±15.48	1,191.01±6,483.03
**MA random**	63	33	1.05±0.21	0.05±0.21	1.49±0.50	2.00±0.00	0.00±0.00
**NI random**	43	22	1.02±0.15	0.00±0.00	1.51±0.50	2.00±0.00	0.02±0.15
**Euclidean<0.1381**							
	**nodes**	**edges**	**<*k*>**	**<*C*>**	**<*l*>**	**<*e*>**	**<*b*>**
**MA**	2990	3066	2.05±1.42	0.17±0.31	13.74±7.48	10.92±11.68	770.42±3,494.26
**NI**	1569	1137	1.45±0.76	0.10±0.28	2.45±1.65	2.69±1.54	2.88±12.17
**MA random**	37	19	1.03±0.16	0.00±0.00	1.51±0.50	2.00±0.00	0.03±0.16
**NI random**	22	11	1.00±0.00	0.00±0.00	1.50±0.50	2.00±0.00	0.00±0.00

Supplemental Figure 2. **Comparison of essential (lethal and sterile knockouts) and non-essential gene fractions in the MA, NI and intersection networks.** (**A**) Percentage of essential genes in each network. (**B**) Contingency table and $\chi 2$ analysis comparing essential (E) and non-essential (NE) genes counts for each network.

A

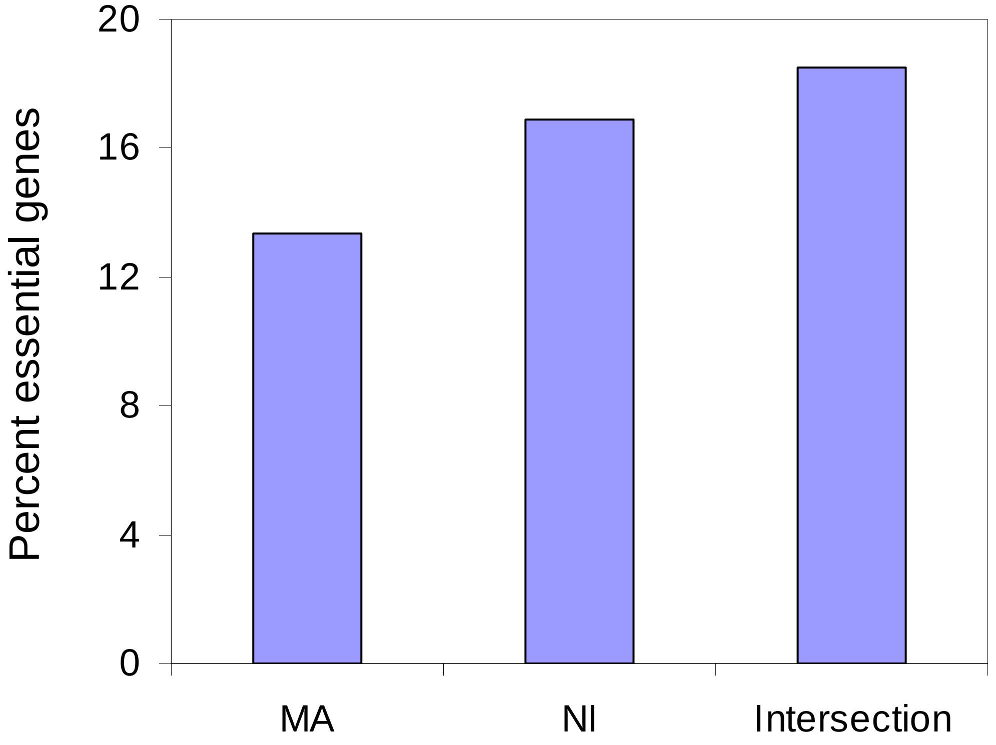

B

	E	NE	totals
**MA**	663	4311	4974
**NI**	685	3365	4050
**Intersection**	353	1552	1905
**totals**	1701	9228	**10929**

χ^2=37.3, df=2, P=8e-09

Supplemental Figure 3. **Control for the effect of the sample size of expression vectors on the overlap between coexpression networks.** The observed overlap, *i.e.* the intersection in terms of the percentage of edges shared between networks, between the MA and NI networks (∩=0.65%) is indicated with the red arrow. A null distribution of *C. elegans* coexpression network overlaps was computed by randomly sampling pairs of expression sets of size (5) from the Kim *et al. C. elegans* gene expression dataset (Science 2001 293: 2087-2092) and computing the overlap between coexpression networks built from these random pair sets. The null distribution of overlaps (average ∩=2.66%) between the random sets of expression data is shown in the black. The difference between the observed and random overlaps is statistically significant (z=40.7 *P*≈0 z test; U=100 *P*=0.02 Mann Whitney U test).

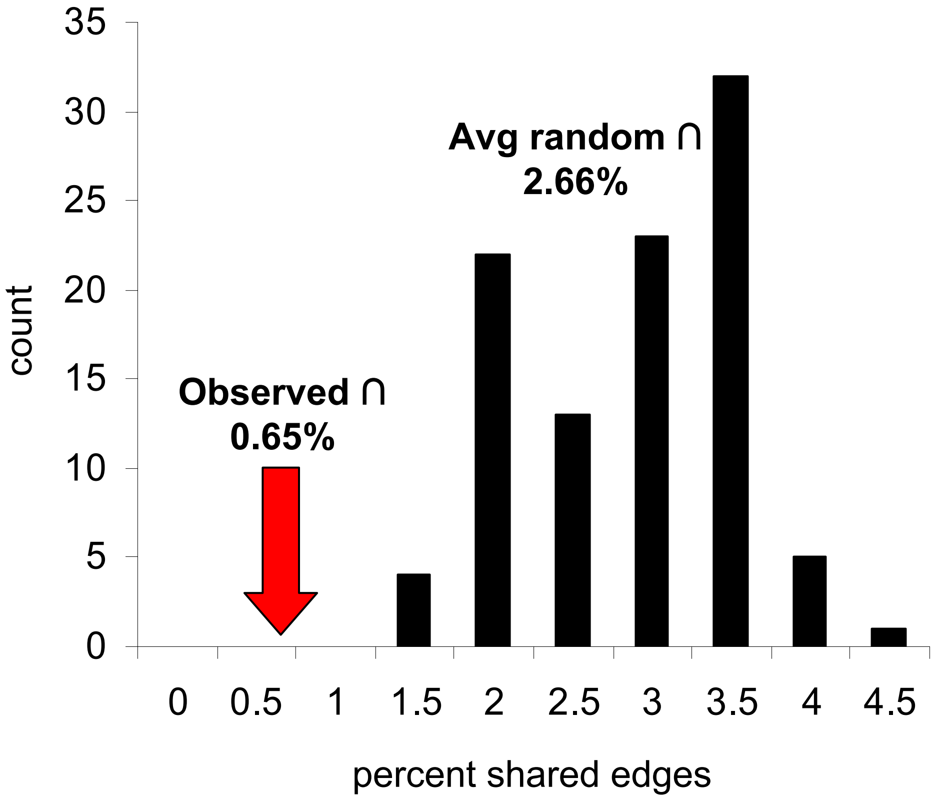

Supplement: Additional file 1 — Supplemental Information. Supplemental Figures 1 – 3 and Supplemental Table 1. [file 1752-0509-2-96-S1.pdf]
